# The Case for Shifting the Rényi Entropy

**DOI:** 10.3390/e21010046

**Published:** 2019-01-09

**Authors:** Francisco J. Valverde-Albacete, Carmen Peláez-Moreno

**Affiliations:** Department of Signal Theory and Communications, Universidad Carlos III de Madrid, 28911 Leganés, Spain

**Keywords:** shifted Rényi entropy, Shannon-type relations, generalized weighted means, Hölder means, escort distributions

## Abstract

We introduce a variant of the Rényi entropy definition that aligns it with the well-known Hölder mean: in the new formulation, the *r*-th order Rényi Entropy is the logarithm of the inverse of the *r*-th order Hölder mean. This brings about new insights into the relationship of the Rényi entropy to quantities close to it, like the information potential and the partition function of statistical mechanics. We also provide expressions that allow us to calculate the Rényi entropies from the Shannon cross-entropy and the escort probabilities. Finally, we discuss why shifting the Rényi entropy is fruitful in some applications.

## 1. Introduction

The suggestive framework for the description and assessment of information transmission that Shannon proposed and co-developed [1,2,3] soon took hold of the mind of a generation of scientists and overflowed its initial field of application, despite the cautions of the inceptor himself [4]. He had independently motivated and re-discovered the Boltzmann description for the thermodynamic entropy of a system with many micro-states [5]. His build-up of the concept starting from Hartley’s measure of information using the nowadays well-known axiomatic approach created a sub-science—perhaps a science—out of three papers. For information scientists, it is difficult to shatter the intellectual chains of Shannon’s entropy [5,6,7,8,9,10,11].

After Shannon’s introduction of his re-purposing of the Boltzmann entropy to analyze communication, many generalizations of it were proposed, among which Rényi’s [12], Hvarda-Charvat-Tsallis’ [13] and Csiszar’s [14] seem to have found the widest echo. Reviews of information measures with different points of view are [14,15].

In this paper we want to contribute to the characterization and popularization of the Rényi entropy as a proper generalization of the Shannon entropy. Rényi’s suggestion was obtained after noticing some limits to the axiomatic approach [16], later better analyzed by Aczel and Daroczny [17]. His critical realisation was that there are more ways to develop the means of the individual surprisals of a collection of events, whereby he resorted to the Kolmogorov-Nagumo theory of the means [18,19,20]. In fact, Kolmogorov had been present in the history Information Theory from foundational issues [18], to punctual clarification [21], to his own devising of a measure of entropy-complexity. The situation concerning the theory of the means at the time is described in [22].

Rényi was quite aware that entropy is a quantity related to the averages of the information function on a probability distribution: let $X∼P_{X}$ be a random variable over a set of outcomes $X=\{x_{i}|1\leq i\leq n\}$ and *pmf* $P_{X}$ defined in terms of the non-null values $p_{i}=P_{X}\left(x_{i}\right)$. The Rényi entropy for *X* is defined in terms of that of $P_{X}$ as $H_{\alpha }\left(X\right)=H_{\alpha }\left(P_{X}\right)$ by a case analysis [12]

(1) $$\begin{array}{cc} \alpha & \neq 1\;H_{\alpha }\left(P_{X}\right)=\frac{1}{1-\alpha }\log\sum_{i=1}^{n}p_{i}^{\alpha }\;\\ \alpha & =1\;\lim_{\alpha \to 1}H_{\alpha }\left(P_{X}\right)=H\left(P_{X}\right) \end{array}$$

where $H\left(P_{X}\right)=-\sum_{i=1}^{n}p_{i}\log p_{i}$ is the Shannon entropy [1,2,3]. Similarly the associated divergence when $Q∼Q_{X}$ is substituted by $P∼P_{X}$ on a compatible support is defined in terms of their *pmf*s $q_{i}=Q_{X}\left(x_{i}\right)$ and $p_{i}=P_{X}\left(x_{i}\right)$, respectively, as $D_{\alpha }\left(X∥Q\right)=D_{\alpha }\left(P_{X}∥Q_{X}\right)$ where

(2) $$\begin{array}{cc} \alpha & \neq 1\;D_{\alpha }\left(P_{X}∥Q_{X}\right)=\frac{1}{\alpha -1}\log\sum_{i=1}^{n}p_{i}^{\alpha }q_{i}^{1-\alpha }\\ \alpha & =1\;\lim_{\alpha \to 1}D_{\alpha }\left(P_{X}∥Q_{X}\right)=D_{KL}\left(P_{X}∥Q_{X}\right). \end{array}$$

and $D_{KL}\left(P_{X}∥Q_{X}\right)=\sum_{i=1}^{n}p_{i}\log\frac{p_{i}}{q_{i}}$ is the Kullback–Leibler divergence [23].

When trying to find the closed form for a generalization of the Shannon entropy that was compatible with all the Faddev axioms but that of linear average, Rényi found that the function $\varphi \left(x\right)=x^{r}$ could be used with the Kolmogorov–Nagumo average to obtain such a new form of entropy. Rather arbitrarily, he decided that the constant should be α=r+1, thus obtaining (1) and (2), but obscuring the relationship of the entropies of order α and the generalized power means.

We propose to shift the parameter in these definitions back to r=α−1 to define the *shifted Rényi entropy of order* *r* the value

$$\begin{array}{cc} {\tilde{H}}_{r}\left(P_{X}\right) & =-\log M_{r}\left(P_{X},P_{X}\right) \end{array}$$

and the *shifted Rényi divergence of order r* the value

$$\begin{array}{cc} {\tilde{D}}_{r}\left(P_{X}∥Q_{X}\right)\; & =\log M_{r}\left(P_{X},\frac{P_{X}}{Q_{X}}\right) \end{array}$$

where $M_{r}$ is the *r-th order weighted generalized power means* or *Hölder means* [24]:

$$\begin{array}{cc} M_{r}\left(\vec{w},\vec{x}\right) & ={\left(\sum_{i=1}^{n}\frac{w_{i}}{\sum_{k}w_{k}}\cdot x_{i}^{r}\right)}^{\frac{1}{r}}. \end{array}$$

In our opinion, this shifted version may be more fruitful than the original. However, since this could be deemed equally arbitrary, in this paper we argue that this statement of the Rényi entropy greatly clarifies its role vis-a-vis the Hölder means, viz. that most of the properties and special cases of the Rényi entropy arise from similar concerns in the Hölder means. We also provide a brief picture of how the theory surrounding the Rényi entropy would be modified with this change, as well as its relationship to some other magnitudes.

## 2. Preliminaries

### 2.1. The Generalized Power Means

Recall that the *generalized power or Hölder mean of order r* is defined as

(3) $$\begin{array}{c} M_{r}\left(\vec{w},\vec{x}\right)={\left(\frac{\sum_{i=1}^{n}w_{i}\cdot x_{i}^{r}}{\sum_{k}w_{k}}\right)}^{\frac{1}{r}}={\left(\sum_{i=1}^{n}\frac{w_{i}}{\sum_{k}w_{k}}\cdot x_{i}^{r}\right)}^{\frac{1}{r}} \end{array}$$

By formal identification, the generalized power mean is nothing but the weighted *f*-mean with $f\left(x\right)=x^{r}$ (see Appendix A). In this paper we use the notation where the weighting vector comes first—rather than the opposite, used in [24]—to align it with formulas in information theory, e.g., divergences and cross entropies. Reference [25] provides proof that this functional mean also has the Properties 1–3 of Proposition A1 and Associativity.

The evolution of $M_{r}\left(\vec{w},\vec{x}\right)$ with *r* is also called the *Hölder path (of an $\vec{x}$)*. Important cases of this mean for historical and practical reasons are obtained by giving values to *r*:

- The (weighted) geometric mean when r=0.
  (4) $$\begin{array}{c} M_{0}\left(\vec{w},\vec{x}\right)=\lim_{r\to 0}M_{r}\left(\vec{w},\vec{x}\right)={\left(\Pi_{i=1}^{n}x_{i}^{w_{i}}\right)}^{\frac{1}{\sum_{k}w_{k}}} \end{array}$$
- The weighted arithmetic mean when r=1.
  $$M_{1}\left(\vec{w},\vec{x}\right)=\sum_{i=1}^{n}\frac{w_{i}}{\sum_{k}w_{k}}\cdot x_{i}$$
- The weighted harmonic mean for r=−1.
  $$M_{-1}\left(\vec{w},\vec{x}\right)={\left(\sum_{i=1}^{n}\frac{w_{i}}{\sum_{k}w_{k}}\cdot x_{i}^{-1}\right)}^{-1}=\frac{\sum_{k}w_{k}}{\sum_{i=1}^{n}w_{i}\cdot \frac{1}{x_{i}}}$$
- The quadratic mean for r=2.
  $$M_{2}\left(\vec{w},\vec{x}\right)={\left(\sum_{i=1}^{n}\frac{w_{i}}{\sum_{k}w_{k}}\cdot x_{i}^{2}\right)}^{\frac{1}{2}}$$
- Finally, the max- and min-means appear as the limits:
  $$\begin{array}{cc} M_{\infty }\left(\vec{w},\vec{x}\right) & =\lim_{r\to \infty }M_{r}\left(\vec{w},\vec{x}\right)=\max_{i=1}^{n}x_{i}\\ M_{-\infty }\left(\vec{w},\vec{x}\right) & =\lim_{r\to -\infty }M_{r}\left(\vec{w},\vec{x}\right)=\min_{i=1}^{n}x_{i} \end{array}$$

They all show the following properties:

**Proposition** **1** (Properties of the weighted power means)**.** *Let $\vec{x},\vec{w}\in {\left(0,\infty \right)}^{n}$ and r,s∈(−∞,∞). Then, the following formal identities hold, where ${\vec{x}}^{r}$ and $\frac{1}{\vec{x}}$ are to be understood entry-wise,*

*1.*  (0- and 1-order homogeneity in weights and values) If $k_{1},k_{2}\in R_{\geq 0}$, then $M_{r}\left(k_{1}\cdot \vec{w},k_{2}\cdot \vec{x}\right)=k_{1}^{0}\cdot k_{2}^{1}\cdot M_{r}\left(\vec{w},\vec{x}\right).$
*2.*  (Order factorization) If r≠0≠s, then $M_{rs}\left(\vec{w},\vec{x}\right)={\left(M_{s}\left(\vec{w},{\left(\vec{x}\right)}^{r}\right)\right)}^{1/r}.$
*3.*  (Reduction to the arithmetic mean) If r≠0, then $M_{r}\left(\vec{w},\vec{x}\right)={\left[M_{1}\left(\vec{w},{\left(\vec{x}\right)}^{r}\right)\right]}^{1/r}$.
*4.*  (Reduction to the harmonic mean) If r≠0, then $M_{-r}\left(\vec{w},\vec{x}\right)={\left[M_{-1}\left(\vec{w},{\left(\vec{x}\right)}^{r}\right)\right]}^{1/r}={\left[M_{r}\left(\vec{w},\frac{1}{\vec{x}}\right)\right]}^{-1}={\left[M_{1}\left(\vec{w},\frac{1}{{\left(\vec{x}\right)}^{r}}\right)\right]}^{-1/r}$.
*5.*  *(Monotonicity in r) Furthermore, $\vec{x}\in {\left[0,\infty \right]}^{n}$ and r,s∈[−∞,∞], then*
  $$\min_{i}x_{i}=M_{-\infty }\left(\vec{w},\vec{x}\right)\leq M_{r}\left(\vec{w},\vec{x}\right)\leq M_{\infty }\left(\vec{w},\vec{x}\right)=\max_{i}x_{i}$$
  *and the mean is a strictly monotonic function of r, that is r<s implies $M_{r}\left(\vec{w},\vec{x}\right)<M_{s}\left(\vec{w},\vec{x}\right)$, unless:*
  - $x_{i}=k$ is constant, in which case $M_{r}\left(\vec{w},\vec{x}\right)=M_{s}\left(\vec{w},\vec{x}\right)=k$.
  - s≤0 and some $x_{i}=0$, in which case $0=M_{r}\left(\vec{w},\vec{x}\right)\leq M_{s}\left(\vec{w},\vec{x}\right)$.
  - 0≤r and some $x_{i}=\infty$, in which case $M_{r}\left(\vec{w},\vec{x}\right)\leq M_{s}\left(\vec{w},\vec{x}\right)=\infty$.
*6.*  *(Non-null derivative) Call ${\tilde{q}}_{r}\left(\vec{w},\vec{x}\right)={\left\{\frac{w_{k}x_{k}^{r}}{\sum_{i}w_{i}x_{i}^{r}}\right\}}_{k=1}^{n}$. Then*
  (5) $$\begin{array}{c} \frac{d}{dr}M_{r}\left(\vec{w},\vec{x}\right)=\frac{1}{r}\cdot M_{r}\left(\vec{w},\vec{x}\right)\ln\frac{M_{0}\left({\tilde{q}}_{r}\left(\vec{w},\vec{x}\right),\vec{x}\right)}{M_{r}\left(\vec{w},\vec{x}\right)} \end{array}$$

**Proof.**  Property 1 follows from the commutativity, associativity and cancellation of sums and products in $R_{\geq 0}$. Property 2 follows from identification in the definition, then Properties 3 and 4 follow from it with s=1 and s=−1 respectively. Property 5 and the special cases in it are well known and studied extensively in [24]. We will next prove property 6

$$\begin{array}{cc} \frac{d}{dr}M_{r}\left(\vec{w},\vec{x}\right) & =\frac{d}{dr}e^{\frac{1}{r}\ln\left(\sum_{k}\frac{w_{k}}{\sum_{i}w_{i}}x_{k}^{r}\right)}=M_{r}\left(\vec{w},\vec{x}\right)\left(\frac{-1}{r^{2}}\ln\left(\sum_{k}\frac{w_{k}}{\sum_{i}w_{i}}x_{k}^{r}\right)+\frac{1}{r}\cdot \frac{\sum_{k}w_{k}x_{k}^{r}\ln x_{k}}{\sum_{i}w_{i}x_{i}^{r}}\right) \end{array}$$

Note that if we call ${\tilde{q}}_{r}\left(\vec{w},\vec{x}\right)={\left\{w_{k}^{\prime }\right\}}_{k=1}^{n}={\left\{\frac{w_{k}x_{k}^{r}}{\sum_{i}w_{i}x_{i}^{r}}\right\}}_{k=1}^{n}$, since this is a probability we may rewrite:

$$\begin{array}{c} \sum_{k}\frac{w_{k}x_{k}^{r}}{\sum_{i}w_{i}x_{i}^{r}}\cdot \ln x_{k}=\sum_{k}w_{k}^{\prime }\ln x_{k}=\ln\left(\prod_{k}x_{k}^{w_{k}^{\prime }}\right)=\ln M_{0}\left({\tilde{q}}_{r}\left(\vec{w},\vec{x}\right),x\right) \end{array}$$

whence

$$\begin{array}{cc} \frac{d}{dr}M_{r}\left(\vec{w},\vec{x}\right) & =M_{r}\left(\vec{w},\vec{x}\right)\left(\frac{1}{r}\cdot \ln M_{0}\left({\tilde{q}}_{r}\left(\vec{w},\vec{x}\right),x\right)-\frac{1}{r}\cdot \ln M_{r}\left(\vec{w},\vec{x}\right)\right)\\ & =\frac{1}{r}\cdot M_{r}\left(\vec{w},\vec{x}\right)\ln\frac{M_{0}\left({\tilde{q}}_{r}\left(\vec{w},\vec{x}\right),\vec{x}\right)}{M_{r}\left(\vec{w},\vec{x}\right)}. \end{array}$$

 □

**Remark** **1.** *The distribution ${\tilde{q}}_{r}\left(\vec{w},\vec{x}\right)$ when $\vec{w}=\vec{x}$ is extremely important in the theory of generalized entropy functions, where it is called a* (shifted) escort distribution (of $\vec{w}$) *[5], and we will prove below that its importance stems, at leasts partially, from this property.*

**Remark** **2.** *Notice that in the case where both conditions at the end of Property 1.5 hold—that is for i≠j we have $x_{i}=0$ and $x_{j}=\infty$—then we have for $r\leq 0,\;M_{r}\left(\vec{w},\vec{x}\right)=0$ and for $0\leq r,M_{r}\left(\vec{w},\vec{x}\right)=\infty$ whence $M_{r}\left(\vec{w},\vec{x}\right)$ has a discontinuity at r=0.*

### 2.2. Rényi’s Entropy

Although the following material is fairly standard, it bears directly into our discussion, hence we introduce it in full.

#### 2.2.1. Probability Spaces, Random Variables and Expectations

Shannon and Rényi set out to find how much information can be gained *on average* by a single performance of an experiment Ω under different suppositions. For that purpose, let $\left(\Omega ,\Sigma_{\Omega },P\right)$ be a measure space, with $\Omega =\{\omega_{1},\ldots ,\omega_{n}\}$ the set of outcomes of a random experiment, $\Sigma_{\Omega }$ the sigma-algebra of this set and measure $P:\Omega \to R_{\geq 0},P\left(\omega_{i}\right)=p_{i},1\leq k\leq n$. We define the support of *P*, as the set of outcomes with positive probability supp (P)={ω∈Ω∣P(ω)>0}.

Let $\left(X,\Sigma_{X}\right)$ be a measurable space with X a domain and $\Sigma_{X}$ its sigma algebra and consider the random variable X:Ω→X, that is, a measurable function so that for each set of $B\in \Sigma_{X}$ we have $X^{-1}\left(B\right)\in \Sigma_{\Omega }$. Then *P* induces a measure $P_{X}$ on $\left(X,\Sigma_{X}\right)$ with $\forall x\in \Sigma_{X},\;P_{X}\left(x\right)=P\left(X=x\right)=P\left(X^{-1}\left(x\right)\right)$, where *x* is an event in $\Sigma_{X}$, and $P_{X}\left(x\right)=\sum_{\omega_{i}\subseteq X^{-1}\left(x\right)}P\left(\omega_{i}\right)$ whereby $\left(X,\Sigma_{X},P_{X}\right)$ becomes a measure space. We will use mostly $X∼P_{X}$ to denote a random variable, instead of its measurable space. The reason for this is that since information measures are defined on distributions, this is the more fundamental notion for us.

Sometimes co-occurring random variables are defined on the same sample space and sometimes on different ones. Hence, we will need another measure space sharing the same measurable space $\left(\Omega ,\Sigma_{\Omega }\right)$ but different measure, $\left(\Omega ,\Sigma_{\Omega },Q\right)$ with $Q\left(\omega_{i}\right)=q_{i}$.

**Remark** **3.** *Modernly, discrete distributions are sets or vectors of non-negative numbers adding up to 1, but Rényi developed his theory for “defective distributions”, that is, with $\sum_{i}P\left(\omega_{i}\right)\neq 1$ which are better described as “positive measures”. In fact, we do not need to distinguish whether P is a probability measure in the (n−1)-simplex $P\in \Delta^{n-1}\Leftrightarrow \sum_{i}P\left(\omega_{i}\right)=1$ or in general a measure $P\in R_{\geq 0}^{n}$ and nothing precludes using the latter to define entropies—while it provides a bit of generalization this is the road we will take below (see [12,26] on using incomplete distributions with $\sum_{i}p_{i}<1$).*

#### 2.2.2. The Approach to Rényi’s Information Functions Based in Postulates

One of the most important applications of the generalized weighted means is to calculate the moments of (non-negative) random variables.

**Lemma** **1.** *Let $X∼P_{X}$ be a discrete random variable. Then ther*-th moment of *Xis:*

(6) $$\begin{array}{c} E_{X}\left\{X^{r}\right\}=\sum_{i}p_{i}x_{i}^{r}={\left(M_{r}\left(P_{X},X\right)\right)}^{r} \end{array}$$

This is the concept that Shannon, and afterwards Rényi, used to quantify information by using the distribution as a random variable (Section 3.3).

The postulate approach to characterize Shannon’s information measures can be found in Appendix B. Analogue generalized postulates lead to Rényi’s information functions, but, importantly, he did not consider normalized measures, that is with $\sum_{k}p_{k}=1$.

We follow [27] in stating the Rényi postulates:

1. The amount of information provided by a single random event $x_{k}$ should be a function of its probability $P_{X}\left(x_{k}\right)=p_{k}$, not its value $x_{k}=X\left(\omega_{k}\right)$, I:[0,1]→I where I⊆R quantifies information.
2. This amount of information should be additive on independent events.
  (7) $$\begin{array}{c} I(p,q)=I(p)+I(q) \end{array}$$
3. The amount of information of a binary equiprobable decision is one bit.
  (8) $$\begin{array}{c} I(1/2)=1 \end{array}$$
4. If different amounts of information occur with different probabilities the total amount of information I is an *average* of the individual information amounts weighted by the probability of occurrence.

These postulates *may* lead to the following consequences:

- Postulates 1 and 2 fix *Hartley’s function* as the single possible amount of information of a basic event
  (9) $$\begin{array}{c} I:[0,1]\to [0,\infty ],p\mapsto I(p)=-k\log p. \end{array}$$
- Postulates 3 fixes the base of the logarithm in Hartley’s formula to 2 by fixing k=1. Any other value k=1/logb fixes b as the base for the logarithm and changes the unit.
- Postulate 4 defines an average amount of information, or *entropy*, properly speaking. Its basic formula is a form of the Kolmogorov–Nagumo formula or *f*-mean (A2) applied to information
  (10) $$\begin{array}{c} H\left(P_{X},\varphi ,I\right)=\varphi^{-1}\left(\;\Sigma_{i=1}^{n}\frac{p_{i}}{\sum_{k}p_{k}}\varphi \left(I\left(p_{i}\right)\right)\right). \end{array}$$
  Thus the “entropy” in Information Theory is, by definition, synonym with “aggregate amount of information”, which departs from its physical etymology, despite the numerous analogies between both concepts.
  It has repeatedly been proven that only two forms of the function φ can actually be used in the Kolmogorov–Nagumo formula that respect the previous postulates [12,26,27]:
  - The one generating Shannon’s entropy:
    (11) $$\begin{array}{c} \varphi (h)=ah+b\;\mathrm{with}\;a\neq 0, \end{array}$$
  - That originally used by Rényi himself:
    (12) $$\begin{array}{c} \varphi \left(h\right)=2^{(1-\alpha )h},\;\mathrm{with}\;\alpha \in \left[-\infty ,\infty \right]\setminus \left\{1\right\}. \end{array}$$

Taking the first form (11) and plugging it into (10) leads to *Shannon’s measure of information*, and taking the second form leads to *Rényi’s measure of information* (1), so we actually have:

**Definition** **1** ([12,26])**.** *The* Rényi entropy of order α *for a discrete random variable $X∼P_{X}$, is*

(13) $$\begin{array}{ccc} & H_{\alpha }\left(P_{X}\right)=\frac{1}{1-\alpha }\log\left(\sum_{i=1}^{n}\frac{p_{i}^{\alpha }}{\sum_{k}p_{k}}\right),\;\alpha \neq 1 & \lim_{\alpha \to 1}H_{\alpha }\left(P_{X}\right)=H\left(P_{X}\right)=-\sum_{i}\frac{p_{i}}{\sum_{k}p_{k}}\log p_{i}, \end{array}$$

*where the fact that Shannon’s entropy is the Rényi entropy when α→1 in* (1) *is found by a continuity argument.*

Rényi also used the postulate approach to define the following quantity:

**Definition** **2** ([12,26])**.** *The* gain of information *or* divergence (between distributions) *when $Y∼P_{Y}$, $P_{Y}\left(y_{i}\right)=q_{i}$ is substituted by $X∼P_{X}$, $P_{X}\left(x_{i}\right)=p_{i}$ being continuous wrt the latter—that is, with supp Y⊆suppX—as*

$$\begin{array}{ccc} D_{\alpha }\left(P_{X}∥P_{Y}\right)=\frac{1}{\alpha -1}\log\sum_{i=1}^{n}p_{i}^{\alpha }q_{i}^{1-\alpha },\;\alpha \neq 1 & \lim_{\alpha \to 1}D_{\alpha }\left(P_{X}∥P_{Y}\right) & =D_{KL}\left(P_{X}∥P_{Y}\right)\; \end{array}$$

*and the fact that Kullback–Leibler’s divergence emerges as the limit when α→1 follows from the same continuity argument as before. Such special cases will not be stated again, as motivated in Section 3.1.*

As in the Shannon entropy case, the rest of the quantities arising in Information Theory can be defined in terms of the generalized entropy and its divergence [23,27].

## 3. Results

### 3.1. The Shifted Rényi Entropy and Divergence

To leverage the theory of generalized means to our advantage, we start with a correction to Rényi’s entropy definition: The investigation into the form of the transformation function for the Rényi entropy (12) is arbitrary in the parameter α that it chooses. In fact, we may substitute in r=α−1 to obtain the pair of formulas:

(14) $$\begin{array}{cccc} \varphi^{\prime }\left(h\right) & =b^{-rh} & \varphi^{\prime -1}\left(p\right) & =\frac{-1}{r}\log_{b}p \end{array}$$

**Definition** **3.** *The shifted Rényi entropy of order r≠0 for a discrete random variable $X∼P_{X}$, is the Kolmogorov–Nagumo $\varphi^{\prime }$-mean* (10) of the information function $I_{*}\left(p\right)=-\ln p$ over the probability values.

(15) $$\begin{array}{cccc} {\tilde{H}}_{r}\left(P_{X}\right) & =\frac{-1}{r}\log_{b}\left(\sum_{i}\frac{p_{i}}{\sum_{k}p_{k}}p_{i}^{r}\right) & \lim_{r\to 0}{\tilde{H}}_{r}\left(P_{X}\right) & =H(P_{X}). \end{array}$$

*Note that:*

- *For r≠0 this is motivated by:*
  $$\begin{array}{cc} {\tilde{H}}_{r}\left(P_{X}\right) & =\frac{-1}{r}\log_{b}\left(\sum_{i}\frac{p_{i}}{\sum_{k}p_{k}}b^{r\log_{b}p_{i}}\right)=\frac{-1}{r}\log_{b}\left(\sum_{i}\frac{p_{i}}{\sum_{k}p_{k}}b^{\log_{b}p_{i}^{r}}\right)=\frac{-1}{r}\log_{b}\left(\sum_{i}\frac{p_{i}}{\sum_{k}p_{k}}p_{i}^{r}\right). \end{array}$$
- *For r=0 we can use the linear mean φ(h)=ah+b with inverse $\varphi^{-1}\left(p\right)=\frac{1}{a}\left(p-b\right)$ as per the standard definition, leading to Shannon’s entropy.*

**Remark** **4.** The base of the logarithm is not important as long as it is maintained in $\varphi^{\prime }\left(\cdot \right)$, $I_{*}\left(\cdot \right)$ and their inverses, hence we leave it implicit. For some calculations—e.g., the derivative below—we explicitly provide a particular basis—e.g., $\log_{e}x=\ln x$.

The shifted divergence can be obtained in the same manner—the way that Rényi followed himself [26].

**Definition** **4.** *The shifted Rényi divergence between two distributions $P_{X}\left(x_{i}\right)=p_{i}$ and $Q_{X}\left(x_{i}\right)=q_{i}$ with compatible support is the following quantity.*

(16) $$\begin{array}{cccc} {\tilde{D}}_{r}\left(P_{X}∥Q_{X}\right) & =\frac{1}{r}\log\sum_{i}\frac{p_{i}}{\sum_{k}p_{k}}{\left(\frac{p_{i}}{q_{i}}\right)}^{r} & \lim_{r\to 0}{\tilde{D}}_{r}\left(P_{X}∥Q_{X}\right) & =D_{KL}\left(P_{X}∥Q_{X}\right). \end{array}$$

Of course, the values of the Rényi entropy and divergence are not modified by this shifting.

**Lemma** **2.** *The Rényi entropy and the shifted Rényi entropy produce the same value, and similarly for their respective divergences.*

**Proof.**  if we consider a new parameter r=α−1 we have:

$$\begin{array}{cc} H_{\alpha }\left(P_{X}\right) & =\frac{1}{1-\alpha }\log\left(\sum_{i=1}^{n}\frac{p_{i}^{\alpha }}{\sum_{k}p_{k}}\right)=\frac{-1}{r}\log\left(\sum_{i=1}^{n}\frac{p_{i}^{r+1}}{\sum_{k}p_{k}}\right)=-\frac{1}{r}\log\left(\sum_{i=1}^{n}\frac{p_{i}}{\sum_{k}p_{k}}p_{i}^{r}\right)={\tilde{H}}_{r}\left(P_{X}\right). \end{array}$$

and similarly for the divergence:

$$\begin{array}{cc} D_{\alpha }\left(P_{X}∥Q_{X}\right) & =\frac{1}{\alpha -1}\log\sum_{i=1}^{n}\frac{p_{i}^{\alpha }q_{i}^{1-\alpha }}{\sum_{k}p_{k}}=\frac{1}{r}\log\sum_{i=1}^{n}\frac{p_{i}^{r+1}q_{i}^{-r}}{\sum_{k}p_{k}}=\frac{1}{r}\log\sum_{i=1}^{n}\frac{p_{i}}{\sum_{k}p_{k}}{\left(\frac{p_{i}}{q_{i}}\right)}^{r}={\tilde{D}}_{r}\left(P_{X}∥Q_{X}\right) \end{array}$$

The Shannon entropy and Kullback–Leibler divergences are clearly the limit cases. □

#### 3.1.1. The Case for Shifting the Rényi Entropy

So what could be the reason for the shifting? First and foremost, it is a re-alignment with the more basic concept of generalized mean.

**Proposition** **2.** *The Shifted Rényi Entropy and Divergence are logarithmic transformations of the generalized power means:*

(17) $$\begin{array}{cc} {\tilde{H}}_{r}\left(P_{X}\right) & =\log\frac{1}{M_{r}\left(P_{X},P_{X}\right)} \end{array}$$

(18) $$\begin{array}{cc} {\tilde{D}}_{r}\left(P_{X}∥Q_{X}\right) & =\log M_{r}\left(P_{X},\frac{P_{X}}{Q_{X}}\right) \end{array}$$

**Proof.**  Simple identification of (15) and (16) in the definition of power mean definitions (3). □

Table 1 lists the shifting of these entropies and their relation both to the means and to the original Rényi definition in the parameter α.

**Remark** **5.** *It is no longer necessary to make the distinction between the case r→0—Shannon’s—and the rest, since the means are already defined with this caveat. This actually downplays the peculiar features of Shannon’s entropy, arising from the geometric mean when $\sum_{i}p_{i}=1$:*

$$\begin{array}{cc} {\tilde{H}}_{0}\left(p_{x}\right) & =\log\frac{1}{M_{0}\left(P_{X},P_{X}\right)}=-\log\left(\prod_{i}p_{i}^{p_{i}}\right)=-\sum_{i}p_{i}\log p_{i} \end{array}$$

*However, the prominence of the Shannon entropy will emerge once again in the context of rewriting entropies in terms of each other (Section 3.2).*

Since the means are properly defined for all r∈[−∞,∞], ${\tilde{H}}_{r}\left(P_{X}\right)$ is likewise properly defined for all r∈[−∞,∞]—and therefore the non-shifted version with α=r+1. This is probably the single strongest argument in favour of the shifting and motivates the following definition.

**Definition** **5** (The Rényi information spectrum)**.** *For fixed $P_{X}$ we will refer to ${\tilde{H}}_{r}\left(P_{X}\right)$ as its* Rényi information spectrum over parameter *r*.

Also, some relationships between magnitudes are clarified in the shifted enunciation with respect to the traditional one, for instance, the relation between the Rényi entropy and divergence.

**Lemma** **3.** *The shifted formulation makes the entropy the self-information with a change of sign:*

(19) $$\begin{array}{cc} {\tilde{H}}_{r}\left(P_{X}\right) & ={\tilde{D}}_{-r}\left(P_{XX}∥P_{X}P_{X}\right). \end{array}$$

**Proof.**  ${\tilde{D}}_{-r}\left(P_{XX}∥P_{X}P_{X}\right)={\tilde{D}}_{-r}\left(P_{X}∥P_{X}P_{X}\right)=\frac{-1}{r}\log\sum_{i}p_{i}{\left(\frac{p_{i}}{p_{i}p_{i}}\right)}^{-r}=\frac{-1}{r}\log\sum_{i}p_{i}{\left(\frac{1}{p_{i}}\right)}^{-r}={\tilde{H}}_{r}\left(P_{X}\right)$. □

Recall that in the common formulation, $H_{\alpha }\left(P_{X}\right)=D_{2-\alpha }\left(P_{X}∥P_{X}P_{X}\right)$ [23].

Another simplification is the fact that the properties of the Rényi entropy and divergence stem from those of the means, inversion and logarithm.

**Proposition** **3** (Properties of the Rényi spectrum of *P_X_*)**.** *Let r,s∈R∪{±∞}, and $P_{X},Q_{X}\in \Delta^{n-1}$ where $\Delta^{n-1}$ is the simplex over the support* supp *X, with cardinal |suppX|=n. Then,*

*1.*  *(Monotonicity) The Rényi entropy is a non-increasing function of the order r.*
  (20) $$\begin{array}{c} s\leq r\Rightarrow {\tilde{H}}_{s}\left(P_{X}\right)\geq {\tilde{H}}_{r}\left(P_{X}\right) \end{array}$$
*2.*  *(Boundedness) The Rényi spectrum ${\tilde{H}}_{r}\left(P_{X}\right)$ is bounded by the limits*
  (21) $$\begin{array}{c} {\tilde{H}}_{-\infty }\left(P_{X}\right)\geq {\tilde{H}}_{r}\left(P_{X}\right)\geq {\tilde{H}}_{\infty }\left(P_{X}\right) \end{array}$$
*3.*  *The entropy of the uniform* pmf *$U_{X}$ is constant over r.*
  (22) $$\begin{array}{c} \forall r\in R\cup \left\{\pm \infty \right\},{\tilde{H}}_{r}\left(U_{X}\right)=\log n \end{array}$$
*4.*  *The Hartley entropy (r=−1) is constant over the distribution simplex.*
  (23) $$\begin{array}{c} {\tilde{H}}_{-1}\left(P_{X}\right)=\log n \end{array}$$
*5.*  *(Divergence from uniformity) The divergence of any distribution $P_{X}$ from the uniform $U_{X}$ can be written in terms of the entropies as:*
  (24) $$\begin{array}{c} {\tilde{D}}_{r}\left(P_{X}∥U_{X}\right)={\tilde{H}}_{r}\left(U_{X}\right)-{\tilde{H}}_{r}\left(P_{X}\right). \end{array}$$
*6.*  *(Derivative of the shifted entropy) The derivative in r of Rényi’s r-th order entropy is*
  (25) $$\begin{array}{c} \frac{d}{dr}{\tilde{H}}_{r}\left(P_{X}\right)=\frac{-1}{r^{2}}{\tilde{D}}_{0}\left({\tilde{q}}_{r}\left(P_{X}\right)∥P_{X}\right)=\frac{-1}{r}\log\frac{M_{0}\left({\tilde{q}}_{r}\left(P_{X}\right),P_{X}\right)}{M_{r}\left(P_{X},P_{X}\right)}, \end{array}$$
  *where ${\tilde{q}}_{r}\left(P_{X}\right)={\left\{\frac{p_{i}p_{i}^{r}}{\sum_{k}p_{k}p_{k}^{r}}\right\}}_{i=1}^{n}$ for r∈R∪{±∞} are the shifted escort distributions.*
*7.*  *(Relationship with the moments of $P_{X}$) The shifted Rényi Entropy of order r is the logarithm of the inverse r-th root of the r-th moment of $P_{X}$.*
  (26) $$\begin{array}{cc} {\tilde{H}}_{r}\left(P_{X}\right) & =-\frac{1}{r}\log E_{P_{X}}\left\{P_{X}^{r}\right\}=\log\frac{1}{\sqrt[{r}]{E_{P_{X}}\left\{P_{X}^{r}\right\}}} \end{array}$$

**Proof.**  Note that properties used in the following are referred to the Proposition they are stated in. Property 1 issues from Property 1.2 and Hartley’s information function being order-inverting or antitone. Since the free parameter *r* is allowed to take values in [−∞,∞], Property 2 follows directly from Property 1. With respect to Property 3, we have, from $U_{X}=1/\left|\mathrm{supp}X\right|=1/n$ and Property A1.3:

$${\tilde{H}}_{r}\left(\frac{1}{n}\right)=-\log M_{r}\left(\frac{1}{n},\frac{1}{n}\right)=-\log\frac{1}{n}=\log n.$$

For Property 4 we have:

$${\tilde{H}}_{-1}\left(P_{X}\right)=-\log{\left(\sum_{i}p_{i}\cdot p_{i}^{-1}\right)}^{-1}=-\log{\left(n\right)}^{-1}=\log n$$

While for Property 5,

$$\begin{array}{cc} {\tilde{D}}_{r}\left(P_{X}∥U_{X}\right) & =\frac{1}{r}\log\left[\sum_{i}p_{i}{\left(\frac{p_{i}}{u_{i}}\right)}^{r}\right]=\frac{1}{r}\log\left[\sum_{i}p_{i}{\left(\frac{p_{i}}{1/n}\right)}^{r}\right]=\frac{1}{r}\log\left[n^{r}\left(\sum_{i}p_{i}p_{i}^{r}\right)\right]\\ & =\log n+\log{\left(\sum_{i}p_{i}p_{i}^{r}\right)}^{1/r}={\tilde{H}}_{r}\left(U_{X}\right)-{\tilde{H}}_{r}\left(P_{X}\right). \end{array}$$

For the third term of Property 6, we have from (17) with natural logarithm, with $P_{X}$ in the role both of $\vec{w}$ and $\vec{x}$

$$\begin{array}{c} \frac{d}{dr}{\tilde{H}}_{r}\left(P_{X}\right)=\frac{d}{dr}\left(-\ln M_{r}\left(P_{X},P_{X}\right)\right)=-\frac{\frac{d}{dr}M_{r}\left(P_{X},P_{X}\right)}{M_{r}\left(P_{X},P_{X}\right)}, \end{array}$$

whence the property follows directly from (5). For the first identity, though, we have:

$$\begin{array}{cc} \frac{d{\tilde{H}}_{r}\left(P_{X}\right)}{dr} & =-\frac{d}{dr}\left[\frac{1}{r}\ln\sum_{i}p_{i}p_{i}^{r}\right]=-\left[\frac{-1}{r^{2}}\ln\sum_{i}p_{i}p_{i}^{r}+\frac{1}{r}\sum_{i}\frac{p_{i}p_{i}^{r}}{\sum_{i}p_{i}p_{i}^{r}}\ln p_{i}\right]. \end{array}$$

If we introduce the abbreviation

(27) $$\begin{array}{cc} {\tilde{q}}_{r}\left(P_{X}\right) & ={\tilde{q}}_{r}\left(P_{X},P_{X}\right)={\left\{{\tilde{q}}_{r}{\left(P_{X}\right)}_{i}\right\}}_{i=1}^{n}={\left\{\frac{p_{i}p_{i}^{r}}{\sum_{k}p_{k}p_{k}^{r}}\right\}}_{i=1}^{n} \end{array}$$

noticing that $\ln\sum_{k}p_{k}p_{k}^{r}=\sum_{i}{\tilde{q}}_{r}{\left(P_{X}\right)}_{i}\ln\left(\sum_{k}p_{k}p_{k}^{r}\right)$, since ${\tilde{q}}_{r}\left(P_{X}\right)$ is a distribution, and factoring out $-1/r^{2}$:

$$\begin{array}{cc} \frac{d{\tilde{H}}_{r}\left(P_{X}\right)}{dr} & =-\frac{1}{r^{2}}\left[-\sum_{i}{\tilde{q}}_{r}{\left(P_{X}\right)}_{i}\ln\left(\sum_{k}p_{k}p_{k}^{r}\right)+r\left(\sum_{i}{\tilde{q}}_{r}{\left(P_{X}\right)}_{i}\ln p_{i}\right)\pm \sum_{i}{\tilde{q}}_{r}{\left(P_{X}\right)}_{i}\ln p_{i}\right]\\ & =-\frac{1}{r^{2}}\left[-\sum_{i}{\tilde{q}}_{r}{\left(P_{X}\right)}_{i}\ln\left(\sum_{k}p_{k}p_{k}^{r}\right)+\left(r+1\right)\sum_{i}{\tilde{q}}_{r}{\left(P_{X}\right)}_{i}\ln p_{i}-\sum_{i}{\tilde{q}}_{r}{\left(P_{X}\right)}_{i}\ln p_{i}\right]\\ & =-\frac{1}{r^{2}}\left[\sum_{i}{\tilde{q}}_{r}{\left(P_{X}\right)}_{i}\ln\frac{p_{i}p_{i}^{r}}{\sum_{k}p_{k}p_{k}^{r}}-\sum_{i}{\tilde{q}}_{r}{\left(P_{X}\right)}_{i}\ln p_{i}\right]=-\frac{1}{r^{2}}\sum_{i}{\tilde{q}}_{r}{\left(P_{X}\right)}_{i}\ln\frac{{\tilde{q}}_{r}{\left(P_{X}\right)}_{i}}{p_{i}} \end{array}$$

and recalling the definition of the shifted divergence we have the result.

For Property 7, in particular, the *probability of any event* is a function of the random variable $P_{X}\left(x_{i}\right)=p_{i}$ whose *r*-th *moment of $P_{X}$* is

(28) $$\begin{array}{c} E_{X}\left\{P_{X}^{r}\right\}=\sum_{i}p_{i}p_{i}^{r}={\left(M_{r}\left(P_{X},P_{X}\right)\right)}^{r} \end{array}$$

The result follows by applying the definition of the shifted entropy in terms of the means. □

**Remark** **6.** *In the preceding proof we have introduced the notion of* shifted escort probabilities *${\tilde{q}}_{r}\left(P_{X}\right)$ acting in the shifted Rényi entropies as the analogues of the* escort probabilities *in the standard definition (see [5] and Section 2.1). This notion of shifted escort probabilities is the one requested by Property 1.6 by instantiation of variables ${\tilde{q}}_{}\left(P_{X}\right)={\tilde{q}}_{}\left(P_{X},P_{X}\right)$. But notice also that ${\left({\tilde{q}}_{r}\left(P_{X}\right)\right)}_{i}=\frac{p_{i}p_{i}^{r}}{\sum_{k}p_{k}p_{k}^{r}}=\frac{p_{i}^{\alpha }}{\sum_{k}p_{k}^{\alpha }}={\left(q_{\alpha }\left(P_{X}\right)\right)}_{i}$ is just the shifting of the traditional escort probabilities [5].*

*Note that for $P_{X}\in R_{\geq 0}^{n}$:*

- *${\tilde{q}}_{0}\left(P_{X}\right)$ is the normalization of $P_{X}$. In fact, $P_{X}\in \Delta^{n-1}$ if and only if we have ${\tilde{q}}_{0}\left(P_{X}\right)=P_{X}$.*
- *${\tilde{q}}_{-1}\left(P_{X}\right)\left(x_{i}\right)={\left|\mathrm{supp}P_{X}\right|}^{-1}$ if $x_{i}\in \mathrm{supp}P_{X}$ and 0 otherwise.*
- *Furthermore, if $P_{X}$ has P maxima (M minima), then ${\tilde{q}}_{\infty }\left(P_{X}\right)$ (${\tilde{q}}_{-\infty }\left(P_{X}\right)$) is an everywhere null distribution but at the indices where the maxima (minima) of $P_{X}$ are situated:*
  $$\begin{array}{cccc} {\tilde{q}}_{\infty }\left(P_{X}\right)\left(x_{i}\right) & =\left\{\begin{array}{cc} \frac{1}{P} & x_{i}\in \arg\max P_{X}\\ 0 & \mathrm{otherwise} \end{array}\right. & {\tilde{q}}_{-\infty }\left(P_{X}\right)\left(x_{i}\right) & =\left\{\begin{array}{cc} \frac{1}{M} & x_{i}\in \arg\min P_{X}\\ 0 & \mathrm{otherwise} \end{array}\right. \end{array}$$

Another important point made clear by this relation to the means is the fact that *all positive measures have a Rényi spectrum*: although so far we conceived the origin of information to be a probability function, nothing precludes applying the same procedure to non-negative, non-normalized quantities with $\sum_{x}f_{X}\left(x\right)\neq 1$, e.g., masses, sums, amounts of energy, etc.

It is well-understood that in this situation Rényi’s entropy has to be slightly modified to accept this procedure. The reason for this is Property 1.1 of the means: generalized means are 1-homogeneous in the numbers being averaged, but 0-homogeneous in the weights. In the Rényi spectrum both these roles are fulfilled by the pmf. Again the escort distributions allow us to analyze the measure:

**Lemma** **4.** *Consider a random variable $X∼M_{X}$ with non-normalized measure $M_{X}\left(x_{i}\right)=m_{i}$ such that $\sum_{i}m_{i}=M\neq 1$. Then the normalized probability measure ${\tilde{q}}_{0}\left(M_{X}\right)={\left\{m_{i}/\sum_{i}m_{i}\right\}}_{i=1}^{n}$ provides a Rényi spectrum that is displaced relative to that of the measure as:*

(29) $$\begin{array}{c} {\tilde{H}}_{r}\left(M_{X}\right)={\tilde{H}}_{r}\left({\tilde{q}}_{0}\left(M_{X}\right)\right)-\log M. \end{array}$$

**Proof.**  $$\begin{array}{cc} {\tilde{H}}_{r}\left({\tilde{q}}_{0}\left(M_{X}\right)\right) & =-\log M_{r}\left({\tilde{q}}_{0}\left(M_{X}\right),{\tilde{q}}_{0}\left(M_{X}\right)\right)=-\frac{1}{r}\log\sum_{i}\frac{m_{i}}{M}{\left(\frac{m_{i}}{M}\right)}^{r}=\log M-\frac{1}{r}\log\sum_{i}\frac{m_{i}}{M}m_{i}^{r}\\ & =\log M-\log M_{r}\left(M_{X},M_{X}\right)=\log M+{\tilde{H}}_{r}\left(M_{X}\right) \end{array}$$

 □

**Remark** **7.** *When M≥1,−logM≤0 with equality for M=1 and that if M<1 then −logM>0. This last was the original setting Rényi envisioned and catered for in the definitions, but nothing precludes the extension provided by Lemma 4. In this paper, although $P_{X}$ can be interpreted as a* pmf *in the formulas, it can also be interpreted as a mass function as in the Lemma above. However, the escort probabilities are always* pmf*s.*

**Figure 1:**
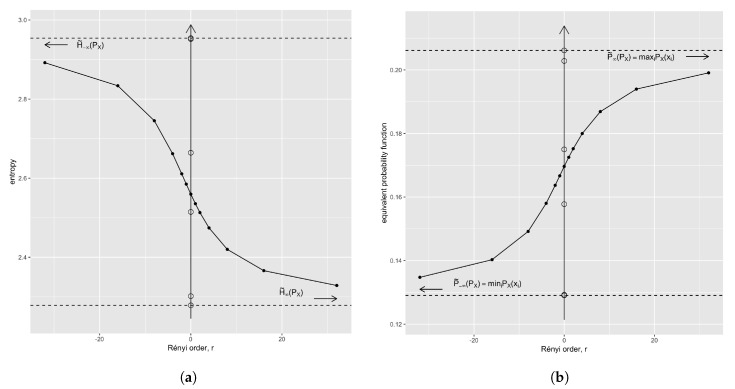
Rényi spectrum (**a**) and equivalent probability function (**b**)—also Hölder path—of ${\tilde{q}}_{0}\left(M_{X}\right)$, the probability distribution of a simple mass measure $M_{X}$ with n=6 (see Section 3.3). The values of the self-information (left) and probability (right) of the original distribution are shown at r=0 also (hollow circles). Only 5 values seem to exist because the maximal information (minimal probability) is almost superposed on a second value.

**Example** **1.** *This example uses the UCB admission data from [28]. We analyze the distribution of admissions with count vector $M_{X}={\left[933\;585\;918\;792\;584\;714\right]}^{⊤}$ and probabilities ${\tilde{q}}_{0}\left(M_{X}\right)\approx {\left[0.21\;0.13\;0.20\;0.17\;0.13\;0.16\right]}^{⊤}$. The names of the departments are not important, due to the symmetry property. Figure 1a shows the Rényi Spectrum extrapolated from a sample of some orders which include r∈{−∞,−1,0,1,∞}.*

#### 3.1.2. Shifting Other Concepts Related to the Entropies

Other entropy-related concepts may also be shifted. In particular, the cross-entropy has an almost direct translation.

**Definition** **6.** *The shifted Rényi cross-entropy of order r∈[−∞,∞] between two distributions $P_{X}\left(x_{i}\right)=p_{i}$ and $Q_{X}\left(x_{i}\right)=q_{i}$ with compatible support is*

(30) $$\begin{array}{cc} {\tilde{X}}_{r}\left(P_{X}∥Q_{X}\right) & =\log\frac{1}{M_{r}\left(P_{X},Q_{X}\right)} \end{array}$$

Note that the case-based definition is redundant: the Shannon cross-entropy appears as ${\tilde{X}}_{0}\left(P_{X}∥Q_{X}\right)=-\log M_{0}\left(P_{X},Q_{X}\right)=-\sum_{i}\frac{p_{i}}{\sum_{k}p_{k}}\log q_{i}$, while for r≠0 we have ${\tilde{X}}_{r}\left(P_{X}∥Q_{X}\right)=-\frac{1}{r}\log\sum_{i}p_{i}{\left(\frac{p_{i}}{q_{i}}\right)}^{r}$ by virtue of the definition of the means again.

Perhaps the most fundamental magnitude is the cross-entropy since it is easy to see that:

**Lemma** **5.** *In the shifted formulation both the entropy and the divergence are functions of the cross-entropy:*

(31) $$\begin{array}{cccc} {\tilde{H}}_{r}\left(P_{X}\right) & ={\tilde{X}}_{r}\left(P_{X}∥P_{X}\right) & {\tilde{D}}_{r}\left(P_{X}∥Q_{X}\right) & ={\tilde{X}}_{-r}\left(P_{X}∥Q_{X}/P_{X}\right) \end{array}$$

**Proof.**  The first equality is by comparison of definitions, while the second comes from:

$$\begin{array}{cc} {\tilde{D}}_{r}\left(P_{X}∥Q_{X}\right) & =\frac{1}{r}\log\sum_{i}p_{i}{\left(\frac{p_{i}}{q_{i}}\right)}^{r}=-\frac{1}{-r}\log\sum_{i}p_{i}{\left(\frac{q_{i}}{p_{i}}\right)}^{-r}={\tilde{X}}_{-r}\left(P_{X}∥Q_{X}/P_{X}\right) \end{array}$$

 □

Note that if we accept the standard criterion in Shannon’s entropy $0\times \log\frac{1}{0}=0\times \infty =0$ then the previous expression for the cross-entropy is defined even if $p_{i}=0$.

### 3.2. Writing Rényi Entropies in Terms of Each Other

Not every expression valid in the case of Shannon’s entropies can be translated into Rényi entropies: recall from the properties of the Kullback–Leibler divergence its expression in terms of the Shannon entropy and cross-entropy. We have:

(32) $$\begin{array}{cc} {\tilde{D}}_{0}\left(P_{X}∥Q_{X}\right) & =-{\tilde{H}}_{0}\left(P_{X}\right)+{\tilde{X}}_{0}\left(P_{X}∥Q_{X}\right), \end{array}$$

but, in general, ${\tilde{D}}_{r}\left(P_{X}∥Q_{X}\right)\neq -{\tilde{H}}_{r}\left(P_{X}\right)+{\tilde{X}}_{r}\left(P_{X}∥Q_{X}\right)$.

However, the shifting sometimes helps in obtaining “derived expressions”. In particular, the (shifted) escort probabilities are ubiquitous in expressions dealing with Rényi entropies and divergences, and allow us to discover the deep relationships between their values for different *r*’s.

**Lemma** **6.** *Let r∈R∪{±∞}, $P_{X}\in \Delta^{n-1}$ where $\Delta^{n-1}$ is the simplex over the support* supp *X. Then,*

(33) $$\begin{array}{cc} {\tilde{H}}_{r}\left(P_{X}\right) & =\frac{1}{r}{\tilde{D}}_{0}\left({\tilde{q}}_{r}\left(P_{X}\right)∥P_{X}\right)+{\tilde{X}}_{0}\left({\tilde{q}}_{r}\left(P_{X}\right)∥P_{X}\right) \end{array}$$

(34) $$\begin{array}{cc} {\tilde{H}}_{r}\left(P_{X}\right) & =\frac{-1}{r}{\tilde{H}}_{0}\left({\tilde{q}}_{r}\left(P_{X}\right)\right)+\frac{r+1}{r}{\tilde{X}}_{0}\left({\tilde{q}}_{r}\left(P_{X}\right)∥P_{X}\right) \end{array}$$

**Proof.**  First, from the definitions of shifted Rényi entropy and cross-entropy and Property 3.6 we have:

$$\begin{array}{c} \frac{-1}{r^{2}}{\tilde{D}}_{0}\left({\tilde{q}}_{r}\left(P_{X}\right)∥P_{X}\right)=\frac{1}{r}\left[{\tilde{H}}_{r}\left(P_{X}\right)-{\tilde{X}}_{0}\left({\tilde{q}}_{r}\left(P_{X}\right)∥P_{X}\right)\right] \end{array}$$

Solving for ${\tilde{H}}_{r}\left(P_{X}\right)$ obtains the first result. By applying (32) to ${\tilde{q}}_{r}\left(P_{X}\right)$ and $P_{X}$ we have:

(35) $$\begin{array}{cc} {\tilde{D}}_{0}\left({\tilde{q}}_{r}\left(P_{X}\right)∥P_{X}\right) & =-{\tilde{H}}_{0}\left({\tilde{q}}_{r}\left(P_{X}\right)\right)+{\tilde{X}}_{0}\left({\tilde{q}}_{r}\left(P_{X}\right)∥P_{X}\right). \end{array}$$

and putting this into (33) obtains the second result.

Another way is to prove it is from the definition of

$$\begin{array}{cc} {\tilde{H}}_{0}\left({\tilde{q}}_{r}\left(P_{X}\right)\right) & =-\sum_{i}\frac{p_{i}p_{i}^{r}}{\sum_{k}p_{k}p_{k}^{r}}\log\frac{p_{i}p_{i}^{r}}{\sum_{k}p_{k}p_{k}^{r}}=\sum_{i}{\tilde{q}}_{r}\left(P_{X}\right)\log\left(\sum_{k}p_{k}p_{k}^{r}\right)-\sum_{i}{\tilde{q}}_{r}\left(P_{X}\right)\log p_{i}^{r+1}\\ & =\log\left(\sum_{k}p_{k}p_{k}^{r}\right)-\left(r+1\right)\sum_{i}{\tilde{q}}_{r}\left(P_{X}\right)\log p_{i}=-r{\tilde{H}}_{r}\left(P_{X}\right)+\left(r+1\right){\tilde{X}}_{0}\left({\tilde{q}}_{r}\left(P_{X}\right)∥P_{X}\right) \end{array}$$

and reorganize to obtain (34). Again inserting the definition of the Shannon divergence in terms of the cross-entropy (35), into (34) and reorganizing we get (33). □

On other occasions, using the shifted version does not help in simplifying expressions. For instance *skew symmetry* looks in the standard case as $D_{\alpha }\left(P_{X}∥Q_{X}\right)=\frac{\alpha }{1-\alpha }D_{1-\alpha }\left(Q_{X}∥P_{X}\right)$, for any 0<α<1 ([23], Proposition 2). In the shifted case we have the slightly more general expression for r≠0:

**Lemma** **7.** *When $Q_{X}$ is substituted by $P_{X}$, both probability distributions, on a compatible support, then:*

(36) $$\begin{array}{c} {\tilde{D}}_{r}\left(P_{X}∥Q_{X}\right)=-\frac{r+1}{r}\cdot {\tilde{D}}_{-(r+1)}\left(Q_{X}∥P_{X}\right) \end{array}$$

**Proof.**  By easy rewriting of the divergence ${\tilde{D}}_{-(r+1)}\left(Q_{X}∥P_{X}\right)$. □

### 3.3. Quantities Around the Shifted Rényi Entropy

On the one hand, the existence of Hartley’s information function (9) ties up information values to probabilities and *vice-versa*. On the other, Rényi’s averaging function and its inverse (14) also transform probabilities into information values and *vice-versa*. In this section we explore the relationship between certain quantities generated by these functions, probabilities and entropies.

#### 3.3.1. The Equivalent Probability Function

Recall that, due to Hartley’s function, from every average measure of information, an equivalent *average* probability emerges. To see this in a more general light, first define the extension to Hartley’s information function to non-negative numbers $I_{*}\left(\cdot \right):\left[0,\infty \right]\to \left[-\infty ,\infty \right]$ as $I_{*}\left(p\right)=-\ln p$. This is one-to-one from [0,∞] and total onto [−∞,∞], with inverse ${\left(I_{*}\right)}^{-1}\left(h\right)=e^{-h}$ for h∈[−∞,∞].

**Definition** **7.** *Let $X∼P_{X}$ with Rényi spectrum ${\tilde{H}}_{r}\left(P_{X}\right)$. Then the* equivalent probability function of ${\tilde{P}}_{r}\left(P_{X}\right)$ *is the Hartley inverse of ${\tilde{H}}_{r}\left(P_{X}\right)$ over all values of r∈[−∞,∞]*

(37) $$\begin{array}{c} {\tilde{P}}_{r}\left(P_{X}\right)={\left(I_{*}\right)}^{-1}\left({\tilde{H}}_{r}\left(P_{X}\right)\right) \end{array}$$

**Remark** **8.** *The equivalent probability function for a fixed probability distribution $P_{X}$ is a function of parameter r—like the Rényi entropy—whose values are probabilities—in the sense that it produces values in [0,1]—but it is* not *a probability distribution.*

*Analogously, due to the extended definition of the Hartley information, this mechanism, when operating on a mass measure $M_{X}$, generates and* equivalent mass function *${\tilde{P}}_{r}\left(M_{X}\right)$, which is* not *a mass measure.*

**Lemma** **8.** *Let $X∼P_{X}$. The* equivalent probability function *${\tilde{P}}_{r}\left(P_{X}\right)$ is the Hölder path of the probability function $P_{X}$ (as a set of numbers) using the same probability function as weights.*

(38) $$\begin{array}{c} {\tilde{P}}_{r}\left(P_{X}\right)=M_{r}\left(P_{X},P_{X}\right) \end{array}$$

**Proof.**  From the definition, using *b* as the basis chosen for the logarithm in the information function.

$$\begin{array}{c} {\tilde{P}}_{r}\left(P_{X}\right)={\left(I_{*}\right)}^{-1}\left({\tilde{H}}_{r}\left(P_{X}\right)\right)=b^{-{\tilde{H}}_{r}\left(P_{X}\right)}=b^{\log_{b}M_{r}\left(P_{X},P_{X}\right)}=M_{r}\left(P_{X},P_{X}\right) \end{array}$$

 □

Note that by Remark 8 these means apply, in general, to sets of non-negative numbers and not only to the probabilities in a distribution, given their homogeneity properties. In the light of Lemma 8, the following properties of the equivalent probability function are a corollary of those of the weighted generalized power means of Proposition 1 in Section 2.1.

**Corollary** **1.** *Let $X∼P_{X}$ be a random variable with equivalent probability function ${\tilde{P}}_{r}\left(P_{X}\right)$. Then:*

*1.*  *For all r∈[−∞,∞], there holds that*
  (39) $$\begin{array}{c} \min_{k}p_{k}={\tilde{P}}_{-\infty }\left(P_{X}\right)\leq {\tilde{P}}_{r}\left(P_{X}\right)\leq \max_{k}p_{k}={\tilde{P}}_{\infty }\left(P_{X}\right) \end{array}$$
*2.*  *If $P_{X}\equiv U_{X}$ the uniform over the same $\mathrm{supp}P_{X}$, then $\forall k,\forall r\in \left[-\infty ,\infty \right],p_{k}={\tilde{P}}_{r}\left(U_{X}\right)=\frac{1}{|\mathrm{supp}P_{X}|}$.*
*3.*  *if $P_{X}\equiv \delta_{X}^{k}$ the Kroneker delta centered on $x_{k}=X\left(\omega_{k}\right)$, then ${\tilde{P}}_{r}\left(\delta_{X}^{k}\right)=u\left(r\right)$ where u(r) is the step function.*

**Proof.**  Claims 1 and 2 issue directly from the properties of the entropies and the inverse to the logarithm. The last claims follows from Remark 2. □

And so, in their turn, the properties of Rényi entropy can be proven from those of the equivalent probability function and Hartley’s generalized information function.

An interesting property might help recovering $P_{X}$ from the equivalent probability function:

**Lemma** **9.** *Let $X∼P_{X}$ be a random variable with equivalent probability function ${\tilde{P}}_{r}\left(P_{X}\right)$. Then: for every $p_{k}$ in $P_{X}$ there exists an $r_{k}\in \left[-\infty ,\infty \right]$ such that $p_{k}={\tilde{P}}_{r_{k}}\left(P_{X}\right)$.*

**Proof.**  This follows from the continuity of the means with respect to its parameters $\vec{w}$ and $\vec{x}$. □

So if we could actually find those values $r_{k},1\leq k\leq n$ which return $p_{k}={\tilde{P}}_{r}\left(P_{X}\right)$ we would be able to retrieve $P_{X}$ by sampling ${\tilde{P}}_{r_{k}}\left(P_{X}\right)$ in the appropriate values $P_{X}={\left\{{\tilde{P}}_{r_{k}}\left(P_{X}\right)\right\}}_{k=1}^{n}$. Since n≥2 we know that at least two of these values are r=±∞ retrieving the value of the highest and lowest probabilities for k=1 and k=n when they are sorted by increasing probability value.

**Example** **2** (Continued)**.** *Figure 1b shows the equivalent probability function of the example in the previous section. The dual monotone behaviour with respect to that of the Rényi spectrum is clearly observable. We have also plotted over the axis at r=0 the original probabilities of the distribution to set it in the context of the properties in Corollary 1 and Lemma 9.*

#### 3.3.2. The Information Potential

In the context of Information Theoretic Learning (ITL) the information potential is an important quantity ([29], Chapter 2).

**Definition** **8.** *Let $X∼P_{X}$. Then the* information potential *${\tilde{V}}_{r}\left(P_{X}\right)$ is*

(40) $$\begin{array}{c} {\tilde{V}}_{r}\left(P_{X}\right)=E_{P_{X}}\left\{P_{X}^{r}\right\}=\sum_{i}\frac{p_{i}}{\sum_{k}p_{k}}p_{i}^{r} \end{array}$$

Note that the original definition of the information potential was presented in terms of parameter α and for distributions with $\sum_{k}p_{k}=1$ in which case $V_{\alpha }\left(P_{X}\right)={\tilde{V}}_{r}\left(P_{X}\right)$. Now, recall the conversion function in (14) $\varphi^{\prime }\left(h\right)=b^{-rh}$. The next lemma is immediate using it on (26).

**Lemma** **10.** *Let $X∼P_{X}$. The information potential is the $\varphi^{\prime }$ image of the shifted Rényi entropy*

(41) $$\begin{array}{c} {\tilde{V}}_{r}\left(P_{X}\right)=\varphi^{\prime }\left({\tilde{H}}_{r}\left(P_{X}\right)\right)=b^{-r{\tilde{H}}_{r}\left(P_{X}\right)} \end{array}$$

**Proof.**  ${\tilde{V}}_{r}\left(P_{X}\right)=b^{-r{\tilde{H}}_{r}\left(P_{X}\right)}=b^{\log_{b}\left(\sum_{i}\frac{p_{i}}{\sum_{k}p_{k}}p_{i}^{r}\right)}=\sum_{i}\frac{p_{i}}{\sum_{k}p_{k}}p_{i}^{r}=E_{P_{X}}\left\{P_{X}^{r}\right\}$ ▯

Incidentally, (28) gives the relation of the information potential and the generalized weighted means.

**Remark** **9.** *The quantity in the right-hand side of* (40) *is also the normalizing factor or* partition function *of the moments of the distribution and, as such, appears explicitly in the definition of the escort probabilities* (27). *Usually other partition functions appear in the estimation of densities based on overt, e.g., maximum entropy [6], or in covert information criteria—e.g., Ising models [5].*

#### 3.3.3. Summary

**Figure 2:**
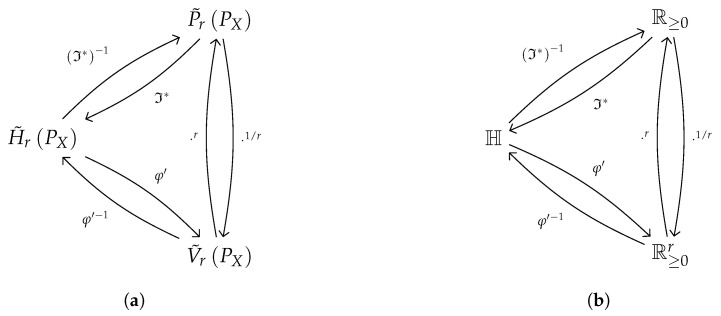
Schematics of relationship between some magnitudes in the text and their domains of definition (see Section 3.4.5). (**a**) Between entropy-related quantities; (**b**) Between entropy-related domains.

Table 2 offers a summary of the quantities mentioned above and their relationships, while the domain diagram in Figure 2 summarizes the actions of these functions to obtain the shifted Rényi entropy. A similar diagram is, of course, available for the standard entropy, using φ with the α parameter.

Note that these quantities have independent motivation: this is historically quite evident in the case of the means [24], and the Rényi information [12] and little bit less so in the case of the information potential which arose in the context of ITL [29], hence motivated by a desire to make Rényi’s entropies more useful. Both quantities are generated from/generate entropy by means of independently motivated functions, Hartley’s transformation (9) and Rényi’s transformation (14), respectively.

Following the original axiomatic approach it would seem we first transform the probabilities into entropies using Hartley’s function and then we use the $\varphi^{\prime }$ function to work out an average of these using the Kolmogorov–Nagumo formula. But due to the formulas for the information potential and the equivalent probability function we know that this is rather a composition of transformations, than a forward backward moving between entropies and probabilities. It is clear that the Hartley function and Rényi’s choice of averaging function are special for entropies, from the postulate approach to their definition.

### 3.4. Discussion

A number of decisions taken in the paper might seem arbitrary. In the following, we try to discuss these issues as well as alternatives left for future work.

#### 3.4.1. Other Reparameterization of the Rényi Entropy

Not only the parameter, but also de sign of the parameter is somewhat arbitrary in the form of (12). If we choose $r^{\prime }=1-\alpha$ another generalization evolves that is, in a sense, symmetrical to the shifted Rényi entropy we have presented above, since $r^{\prime }=-r$. This may be better or worse for the general formulas describing entropy, etc., but presents the problem that it no longer aligns with Shannon’s original choice of sign. The r=0 order Rényi entropy would actually be Boltzmann’s, negative entropy or *negentropy* [30] and perhaps more suitable for applications in Thermodynamics [5].

Yet another formulation suggests the use of α=1/2, equivalently r=−1/2 as the origin of the parameter [31]. From our perspective, this suggests that the origin of the Rényi entropy can be chosen adequately in each application.

#### 3.4.2. Rényi Measures and the Means

The usefulness of the (weighted) means in relation to information-theoretic concerns was already noted and explored in [32]. However, the relationship is not in there explicitly set out in terms of the identity of the Rényi entropies and logarithmic, weighted means of probabilities but rather as a part of establishing bounds for different quantities for discrete channel characterization.

A more direct approach is found in [33] that, inspired by [32], decides to generalize several results from there and other authors concerning the Rényi entropies, divergences and the Rényi centers of a set of distributions. Unlike our proposal, this deep work adheres to the standard definition of Rényi entropies of order α and avoids the issue of negative orders. The focus here is in coding and channel theorems, while ours is a re-definition of the mathematical concept to make similarities with weighted means transparent, yet evident.

#### 3.4.3. Other Magnitudes around the Rényi Entropy

Sometimes the *p*-norm is used as a magnitude related to the Rényi entropy much as the information potential [29] or directly seeing the relationship with the definition [5].

**Definition** **9.** *For a set of non-negative numbers $\vec{x}={\left[x_{i}\right]}_{i=1}^{n}\in {\left[0,\infty \right)}^{n}$ the p-norm, with 0≤p≤∞ is*

(42) $$\begin{array}{c} ∥\vec{x}{∥}_{p}={\left(\sum_{i}x_{i}^{p}\right)}^{\frac{1}{p}} \end{array}$$

A more general definition involves both positive and negative components for $\vec{x}$, as in normed real spaces, but this is not relevant to our purposes for non-negative measures.

The *p*-norm has the evident problem that it is only defined for positive *p* whereas (14) proves that negative orders are meaningful and, indeed, interesting. A prior review of results for the negative orders can be found in [23].

We believe this is yet one more advantage of the shifting of the Rényi order: that the relation with the equivalent probability function and the information potential—the moments of the distribution—are properly highlighted.

#### 3.4.4. Redundancy of the Rényi Entropy

Lemma 6 proves that Rényi entropies are very redundant in the sense that given its value for a particular $r_{0}$ the rest can be written in terms of those entropies with different, but systematically related, *r* order (see Section 3.4.4).

In particular, Equations (33) and (34) in Lemma 6, and (31) in Lemma 5 allow us to use a good estimator of Shannon’s entropy to estimate the Rényi entropies and related magnitudes for all orders, special or not. Three interesting possibilities for this rewriting are:

- *That everything can be written in terms of r=0, e.g., in terms of Shannon’s entropy.* This is made possible by the existence of estimators for Shannon’s entropy and divergence.
- *That everything can be written in terms of a finite r≠0, e.g., r=1.* This is possible by means of Properties 1.3 and 1.4 of the generalized power means. The work in [29] is pointing this way (perhaps including also r=−1, aka Hartley’s) capitalizing on the fact that Rényi’s entropy for data is well estimated for r=1, equivalently α=2 ([29], Section 2.6).
- *That everything can be written in terms of the extreme values of the entropy, e.g., r=±∞.* This is suggested by Properties 3.1 and 3.2. Supposing we had a way to estimate either ${\tilde{H}}_{-\infty }\left(P_{X}\right)$ or ${\tilde{H}}_{\infty }\left(P_{X}\right)$. Then by a divide-and-conquer type of approach it would be feasible to extract all the probabilities of a distribution out of its Rényi entropy function.

#### 3.4.5. The Algebra of Entropies

Technically, the completed non-negative reals $R_{\geq 0}$, where the means are defined, carry a complete positive semifield structure [34]. This is an algebra similar to a real-valued field but the inverse operation to addition, e.g., subtraction, is missing.

There are some technicalities involving writing the results of the operations of the extremes of the semifields—e.g., multiplication of 0 and *∞*—and this makes writing closed expressions for the means with extreme values of $\vec{w}$ or $\vec{x}$ complicated. A sample of this is the plethora of conditions on Property 1.5. An extended notation, pioneered by Moreau [35], is however capable of writing a closed expression for the means [36].

Furthermore, taking (minus) logarithms and raising to a real power are isomorphism of semifields, so that the Rényi entropies inhabit a different positive semifield structure [36]. The graph of these isomorphic structures can be seen in Figure 2b. This means that some of the intuitions about operating with entropies are misguided. We believe that failing to give a meaning to the Rényi entropies with negative orders might have been caused by this.

#### 3.4.6. Shifted Rényi Entropies on Continuous Distributions

The treatment we use here may be repeated on continuous measures, but the definitions of Shannon [10,21] and Rényi [26] entropies in such case run into technical difficulties solved, typically, by a process of discretization [27].

Actually we believe that the shifting would also help in this process: a form for the generalized weighted continuous means was long ago established [20] and technically solved by a change of concept and Lebesgue–Stieltjes integration instead of summation ([24], Ch. VI).

Our preliminary analyses show that the relationship with the means given by (17) also holds, and this would mean that the shifting—in aligning the Rényi entropies with the (generalized weighted) continuous means—leverages the theoretical support of the latter to sustain the former.

**Definition** **10** (Continuous weighted *f*-mean)**.** *Let Φ(ξ) be a measure and let f be a monotonic function of ξ with inverse $f^{-1}$. Then a continuous version of* (A2) *is:*

$$\begin{array}{cc} M_{f}\left(\Phi ,\xi \right) & =f^{-1}\left\{\int f(\xi )d\Phi (\xi )\right\} \end{array}$$

*understood as a Lebesgue–Stieltjes integral.*

This definition was already proposed by De Finetti [20] based upon the works of Bonferroni and Kolmogorov and thoroughly developed in ([24], Ch. VI) in connection to the discrete means. With $f\left(x\right)=x^{r}$ the continuous Hölder means $M_{r}\left(\Phi ,\xi \right)$ appear. Furthermore De Finetti found ([20], #8) that the form of the *f* continuous, monotone function *f* must be

$$\begin{array}{ccc} f(x) & =a\int \gamma (x)dx+b & \mathrm{for}\;\mathrm{arbitrary}\;a,b(a\neq 0) \end{array}$$

similar to what Rényi found later for the Shannon entropy.

It is easy to see that an analogue definition of the shifted Rényi entropy but for a continuous probability density $p_{X}$ with $dp_{X}\left(x\right)=p_{X}\left(x\right)dx$ [5,27] is

(43) $$\begin{array}{c} \tilde{h}\left(p_{X}\right)=\frac{-1}{r}\log\int p_{X}\left(x\right)p_{X}^{r}\left(x\right)dx=-\log M_{r}\left(p_{X},p_{X}\right) \end{array}$$

again with the distribution acting as weight and averaged quantity. Compare this to one of the standard forms of the *differential Rényi entropy* [23]:

$$\begin{array}{c} h\left(p_{X}\right)=\frac{1}{1-\alpha }\ln\int p_{X}^{\alpha }\left(x\right)dx \end{array}$$

The investigation of the properties of (43) is left pending for future work, though.

#### 3.4.7. Pervasiveness of Rényi Entropies

Apart from the evident applications to signal processing and communications [29], physics [5] and cognition [11], the Rényi entropy is a measure of diversity in several disciplines [37]. We believe that, if its applicability comes from the same properties stemming from the means that we have explored in this paper as applied to positive distributions—e.g., of wealth in a population, or energy in a community—, then the expression to be used is (29).

## 4. Conclusions

In this paper we have advocated for the shifting of the traditional Rényi entropy order from a parameter α to r=α−1. The shifting of the Rényi entropy and divergence is motivated by a number of results:

- It aligns them with the power means and explains the apparition of the escort probabilities. Note that the importance of the escort probabilities is justified independently of their link to the means in the shifted version of entropy [5].
- It highlights the Shannon entropy r=0 in the role of the “origin” of entropy orders, just as the geometric means is a particular case of the weighted averaged means. This consideration is enhanced by the existence of a formula allowing us to rewrite every other order as a combination of Shannon entropies and cross entropies of escort probabilities of the distribution.
- The shifting of the Rényi entropy aligns it with the moments of the distribution, thus enabling new insights into the moments’ problem.
- It makes the relation between the divergence and the entropy more “symmetrical”.
- It highlights the “information spectrum” quality of the Rényi entropy measure for fixed $P_{X}$.

The shifting might or might not be justified by applications. If the concept of the means is relevant in the application, we recommend the shifted formulation.

## Figures and Tables

**Table 1:** Relation between the most usual weighted power means, Rényi entropies and shifted versions of them.

Mean Name	Mean $M_{r}\left(\vec{w},\vec{x}\right)$	Shifted Entropy ${\tilde{H}}_{r}\left(P_{X}\right)$	Entropy Name	α	*r*
Maximum	$\max_{i}x_{i}$	${\tilde{H}}_{\infty }=-\log\max_{i}p_{i}$	min-entropy	*∞*	*∞*
Arithmetic	$\sum_{i}w_{i}x_{i}$	${\tilde{H}}_{1}=-\log\sum_{i}p_{i}^{2}$	Rényi’s quadratic	2	1
Geometric	$\Pi_{i}x_{i}^{w_{i}}$	${\tilde{H}}_{0}=-\sum_{i}p_{i}\log p_{i}$	Shannon’s	1	0
Harmonic	${\left(\sum_{i}w_{i}\frac{1}{x_{i}}\right)}^{-1}$	${\tilde{H}}_{-1}=\log n$	Hartley’s	0	−1
Minimum	$\min_{i}x_{i}$	${\tilde{H}}_{-\infty }=-\log\min_{i}p_{i}$	max-entropy	−∞	−∞

**Table 2:** Quantities around the shifted Rényi entropy of a discrete distribution $P_{X}$.

Quantity in Terms of…	Rényi Entropy	Gen. Hölder Mean	Information Potential	Distribution
Rényi entropy	${\tilde{H}}_{r}\left(P_{X}\right)$	$-\log M_{r}\left(P_{X},P_{X}\right)$	$\frac{-1}{r}\log{\tilde{V}}_{r}\left(P_{X}\right)$	$\frac{-1}{r}\log\left(\sum_{i}\frac{p_{i}}{\sum_{k}p_{k}}p_{i}^{r}\right)$
Gen. Hölder mean	$\exp(-{\tilde{H}}_{r}\left(P_{X}\right))$	$M_{r}\left(P_{X},P_{X}\right)$	${\left({\tilde{V}}_{r}\left(P_{X}\right)\right)}^{\frac{1}{r}}$	${\left(\sum_{i}\frac{p_{i}}{\sum_{k}p_{k}}p_{i}^{r}\right)}^{\frac{1}{r}}$
Information potential	$\exp(-r{\tilde{H}}_{r}\left(P_{X}\right))$	$M_{r}{\left(P_{X},P_{X}\right)}^{r}$	${\tilde{V}}_{r}\left(P_{X}\right)=E_{P_{X}}\left\{P_{X}^{r}\right\}$	$\sum_{i}\frac{p_{i}}{\sum_{k}p_{k}}p_{i}^{r}$

## Appendix A. The Kolmogorov-Mean and the Kolmogorov–Nagumo Formula

The following is well known since [18,19,20,24].

**Definition** **A1.**
*Given an invertible real function f:R→R the Kolmogorov–Nagumo mean of a set of non-negative numbers $\vec{x}={\left[x_{i}\right]}_{i=1}^{n}\in {\left[0,\infty \right)}^{n}$ is*

(A1) $$\begin{array}{c} KN_{f}\left(\vec{x}\right)=f^{-1}\left(\sum_{i=1}^{n}\frac{1}{n}f\left(x_{i}\right)\right). \end{array}$$

Definition A1 is an instance of the following formula to work out the *weighted f-mean* with a set of finite, non-negative weights, $\vec{w}\in \left[0,\infty \right)$

(A2) $$\begin{array}{c} M_{f}\left(\vec{w},\vec{x}\right)=f^{-1}\left(\sum_{i=1}^{n}\frac{w_{i}}{\sum_{k}w_{k}}f\left(x_{i}\right)\right). \end{array}$$

Our interest in (A2) lies in the fact that Shannon’s and Rényi’s entropies can be seen as special cases of it, which makes its properties especially interesting.

**Proposition** **A1** (Properties of the Kolmogorov–Nagumo means)**.** *Let $\vec{x},\vec{w}\in {\left[0,\infty \right)}^{n}$. The following conditions hold if and only if there is a strictly monotonic and continuous function f such that* (A1) *holds.*

1. Continuity and strict monotonicity in all coordinates.
2. (Symmetry or permutation invariance) *Let σ be a permutation, then $M_{f}\left(\vec{w},\vec{x}\right)=M_{f}\left(\sigma \left(\vec{w}\right),\sigma \left(\vec{x}\right)\right)$.*
3. (Reflexivity) *The mean of a series of constants is the constant itself:*
  $$M_{f}\left(\vec{w},{\left\{k\right\}}_{i=1}^{n}\right)=k$$
4. (Blocking) *The computation of the mean can be split into computations of equal size sub-blocks.*
5. (Associativity) *Replacing a k-subset of the x with their partial mean in the same multiplicity does not change the overall mean.*

For a minimal axiomatization, Blocking and Associativity are redundant. A review of the axiomatization of these and other properties can be found in [22].

## Appendix B. The Approach to Shannon’s Information Functions Based in Postulates

It is important to recall that Shannon set out to define the *amount of information*, discarding any *notion of information* itself. Both concepts should be distinguished clearly for methodological reasons, but can be ignored for applications that deal only with quantifying information.

Recall the Faddeev postulates for the generalization of Shannon’s entropy ([26], Chap. IX. §2):

1. The *amount of information* H(P) of a sequence $P={\left[p_{k}\right]}_{k=1}^{n}$ of *n* numbers is a symmetric function of this set of values $H\left(P\right)=H\left(\sigma \left(P\right)\right)=H\left({\left\{p_{k}\right\}}_{k=1}^{n}\right)$, where σ is any permutation of *n*-elements.
2. H({p,1−p}) is a continuous function of p,0≤p≤1.
3. $H(\left\{\frac{1}{2},\frac{1}{2}\right\})=1$.
4. The following relation holds:
  (A3) $$\begin{array}{c} H\left(\left\{p_{1},p_{2},\ldots ,p_{n}\right\}\right)=H\left(\left\{p_{1}+p_{2},\ldots ,p_{n}\right\}\right)+\left(p_{1}+p_{2}\right)H\left(\left\{\frac{p_{1}}{p_{1}+p_{2}},\frac{p_{2}}{p_{1}+p_{2}}\right\}\right) \end{array}$$

These postulates lead to Shannon’s entropy for $X∼P_{X}$ with binary logarithm [26]

(A4) $$\begin{array}{c} H\left(P_{X}\right)=E_{P_{X}}\left\{-\log P_{X}\right\}=-\sum_{k}p_{k}\log p_{k} \end{array}$$
